# Application Value and Safety Analysis of Warfarin, Rivaroxaban, and Dabigatran Ester in Elderly Patients With Atrial Fibrillation

**DOI:** 10.1002/clc.70014

**Published:** 2024-09-09

**Authors:** Cheng Chen, Qing Tian, Chong Cheng, Xixin Ji, Mengyun Feng, Huiqun Tan, Qian Zhou

**Affiliations:** ^1^ Department of Geriatrics Huangshi Central Hospital, Affiliated Hospital of Hubei Polytechnic University Huangshi Hubei China; ^2^ Department of General Practice Huangshi Central Hospital, Affiliated Hospital of Hubei Polytechnic University Huangshi Hubei China

**Keywords:** anticoagulation, atrial fibrillation, drug safety, elderly patient

## Abstract

**Background:**

This study aimed to evaluate the application value and safety of Warfarin, Rivaroxaban, and Dabigatran in elderly patients with atrial fibrillation.

**Methods:**

A total of 180 elderly patients with atrial fibrillation admitted to our hospital were retrospectively analyzed. According to their anticoagulant treatment regimen, patients were divided into three groups: Warfarin (57 cases), Rivaroxaban (61 cases), and Dabigatran (62 cases). General demographic information was collected, and coagulation function indicators—including fibrinogen (FIB), thrombin time (PT), activated partial thrombin time (APTT), and D‐dimer (D‐D)—as well as liver function indexes—including total bilirubin (TbiL), alkaline phosphatase (ALP), aspartate aminotransferase (AST), and alanine transferase (ALT)—were compared before and after 4 weeks of treatment.

**Results:**

There were no significant differences in demographic characteristics such as gender, age, body mass index, or disease course among the three groups. The total effective rate in the Warfarin group (84.21%) was significantly lower than in the Rivaroxaban (98.36%) and Dabigatran (96.77%) groups (*p* < 0.05). However, there was no significant difference in the total effective rate between the Rivaroxaban and Dabigatran groups (*p* > 0.05). Additionally, no significant differences were found in the effects of the three drugs on coagulation function, liver function, or the incidence of bleeding (*p* = 0.052).

**Conclusion:**

Warfarin, Rivaroxaban, and Dabigatran can effectively prevent thrombosis in elderly patients with atrial fibrillation, with Rivaroxaban and Dabigatran showing superior effectiveness. All three drugs demonstrated similar low rates of bleeding events and had no significant impact on coagulation and liver function.

## Graphical abstract

The anticoagulants Warfarin, Rivaroxaban, and Dabigatran are efficacious in preventing thrombosis among elderly individuals with atrial fibrillation, with Rivaroxaban and Dabigatran demonstrating superior efficacy. These medications exhibit comparable and minimal incidences of bleeding complications, and they exert no significant impact on coagulation profiles or liver function.

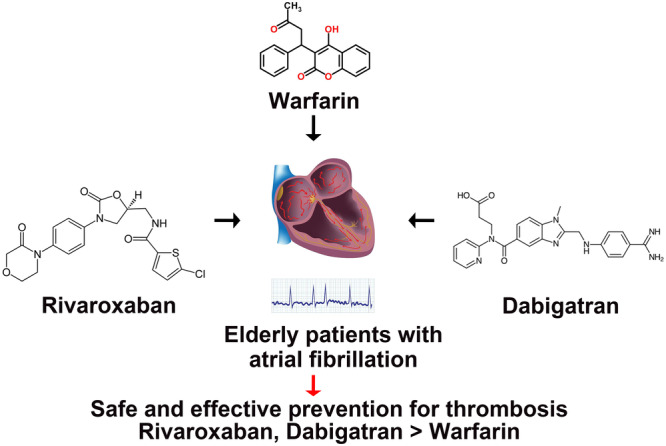

**Abbreviations**
ALP: alkaline phosphatase
ALT: alanine transferase
APTT: activated partial thrombin time
AST: aspartate aminotransferase
D‐D: D‐dimer
FIB: fibrinogen
NOAC: nonvitamin K antagonist oral anticoagulants
PT: thrombin time
TbiL: total bilirubin

## 1 Background

Atrial fibrillation is one of the most common persistent tachyarrhythmias. The occurrence of atrial fibrillation is related to age [1]. Studies have indicated that atrial fibrillation in the elderly population accounts for 70% of all patients with atrial fibrillation [2]. In addition, the incidence of atrial fibrillation in the 80‐year‐old population is as high as 8.8% [3]. A large population‐based study has shown that at least 1/4 to 1/3 of patients with atrial fibrillation aged 75 years or older have an ischemic stroke, and cardiogenic stroke accounts for at least 80% [4]. Therefore, the diagnosis and treatment of atrial fibrillation in the elderly occupy an important position in basic cardiovascular diagnosis and treatment. Anticoagulation therapy is an important means to prevent stroke in patients with atrial fibrillation, and it can also benefit elderly patients [5]. However, due to the complexity of diseases in the elderly, coexisting risks of thrombosis and bleeding, combined with multidrug therapy, often accompanied by cognitive decline and weakness, greatly affecting the safety of anticoagulant drugs used in elderly patients, elderly patients with atrial fibrillation have a low anticoagulant rate and an even lower rate of compliance [6]. Therefore, it is very important to seek a convenient and feasible standardized anticoagulation management scheme for the anticoagulation treatment of elderly patients with atrial fibrillation.

Warfarin as a commonly used drug to prevent thromboembolism in clinical practice, belonging to the vitamin K antagonist class anticoagulant drugs interferes with vitamin K participation in coagulation factors Ⅱ, Ⅷ, Ⅹ in the liver synthesis process [7]. But its effect is slow, the general treatment of about a week before the efficacy of stable. Moreover, the therapeutic effect was greatly affected by genetic factors and environmental factors, and the bleeding situation after the medication was significantly affected by individual differences [8]. Compared with Warfarin, nonvitamin K antagonist oral anticoagulants (NOAC) anticoagulant therapy, such as Dabigatran and Rivaroxaban, was associated with a reduced risk of stroke in certain age and sex subgroups [9].

Rivaroxaban does not require frequent monitoring during use, is less influenced by other food and genetic factors, and has no effect on liver function [10]. Dabigatran ester can directly block the coagulation cascade reaction by binding type Ⅱa and free type Ⅱa [11]. However, there is still a lack of clinical reports on which of the above‐mentioned regimens is more advantageous in the treatment of elderly patients with atrial fibrillation. Based on this, this study aimed to compare the efficacy and drug safety of three anticoagulant regimens, Warfarin, Rivaroxaban, and Dabigatran, in elderly patients with atrial fibrillation, to provide evidence for clinical prevention and treatment of elderly patients with atrial fibrillation.

## 2 Materials and Methods

### 2.1 Subjects

A total of 180 elderly patients with atrial fibrillation who were treated in our hospital from June 2020 to June 2022 were included in this study. According to the anticoagulant treatment regimen, the patients were divided into the Warfarin group (57 cases), Rivaroxaban group (61 cases), and Dabigatran group (62 cases). This study was approved by the ethical review committee of Huangshi Central Hospital. Written informed consent was obtained from all the patients regarding their participation in the study. This study was conducted in compliance with the Helsinki Declaration.

Patient inclusion criteria: (1) Patients over 60 years old; (2) patients meeting the atrial fibrillation thrombotic risk score (CHADS2) [12] ≥ 2 score; (3) Atrial fibrillation bleeding risk score(HAS‐BLED) [13] < 3 score. Exclusion criteria: (1) Patients with severely abnormal liver and kidney function; (2) malignant tumor or blood system disease; (3) patients with cardiovascular and cerebrovascular events; (4) massive hemorrhage or severe trauma within the past 6 months; (5) contraindications of anticoagulation; (6) history of mitral stenosis, mechanical valve, pulmonary embolism, renal vein thrombosis, and so on. (7) taking other anticoagulant drugs; (8) patients with high bleeding risk factors such as thrombocytopenia, puncture, trauma, or hemorrhagic disease.

### 2.2 Anticoagulant Therapy

Warfarin Group was awarded to Warfarin (manufacturer: Shanghai Xinyi Jiufu Pharmaceutical Co., LTD.; Batch number: 18151204E) orally, with an initial dose of 2 mg/day and international normalized ratio (INR) of 2.0–3.0, for 6 months.

Rivaroxaban group to Rivaroxaban (manufacturer: Bayer GMBH; Batch number: BXHVX31) Orally, 15 mg/time, once/day [14]. At the same time, the dosage should be adjusted timely according to the actual situation of the patients. For patients over 75 years old with a body weight of less than 45 kg, 10 mg/time should be taken daily for 6 months.

The Dabigatran group was given Dabigatran ester (manufacturer: Boehringer Ingelheim GMBH; Batch number: 801623/B12322) Orally, 110 mg/time, twice/day, lifetime medication.

### 2.3 Clinical Efficacy Evaluation

We considered the occurrence of embolic events within 6 months of the start of treatment as ineffective, and the absence of embolic events as effective.

### 2.4 Evaluation of Coagulation and Liver Function

A total of 5 mL of elbow venous blood was extracted from the patient before anticoagulant therapy and 4 weeks after treatment. After centrifugation, the changes in fibrinogen (FIB), thrombin time (PT), activated partial thrombin time (APTT), and D‐dimer (D‐D). Coagulation function was measured by CA‐7000 automatic blood coagulation analyzer manufactured by Sysmex in Japan and The kit was produced by Shanghai Yuyan Scientific Instrument Co. LTD. A total of 5 mL of fasting venous blood was extracted from patients before anticoagulant therapy and at the 4th week after treatment, centrifuged and sent for examination to measure total bilirubin (TbiL), alkaline phosphatase (ALP), aspartate aminotransferase (AST), and alanine transferase (ALT) indexes.

### 2.5 Evaluation of Drug Safety

Patients were followed up for 12 months by phone, WeChat, and medical records after rehospitalization. The information on embolic events and bleeding events was recorded. Embolism events include myocardial infarction and cerebral infarction; Bleeding events include black stools, hematuria, and bleeding elsewhere (nose bleeding, gum bleeding, and subcutaneous bleeding).

### 2.6 Statistical Analysis

Statistical analysis was performed using SPSS21.0 software. The normality of continuous variables was tested by the Shapiro‐Wilk test as well as the graphical illustration of histograms and Q‐Q plots. Normally distributed measurement data were expressed as mean ± standard deviation (SD), while nonnormally distributed measurement data were expressed as median (interquartile range), and the comparisons were examined by Student‐*t* test and Mann–Whitney test (nonparametric distribution). The categorical data were expressed as *n* (%), and the differences between the two groups were examined by chi‐square analysis or Fisher's Exact Test. The statistical significance level was set at 0.05 for a two‐sided test.

## 3 Results

### 3.1 General Data

The Warfarin group included 27 males and 30 females, with a mean age of (71.39 ± 11.19) years, ranging from 62 to 82 years. The Rivaroxaban group included 29 males and 32 females, with an average age of (71.48 ± 11.08) years old (63–83 years old), and the Dabigatran group included 28 males and 34 females, with an average age of (71.23 ± 11.23) years old (62–83 years old). There was no significant difference in the general data among the three groups (*p* > 0.05) (Table 1).

**Table 1:** Comparison of general conditions among the three groups.

Index	Warfarin group (*n* = 57)	Rivaroxaban group (*n* = 61)	Dabigatran group (*n* = 62)	*F*/*χ* ^2^	*p*
Age (year)	71.39 ± 11.19	71.42 ± 11.08	71.48 ± 11.08	0.001	0.999^a^
Gender				0.086	0.957^b^
Male	27	29	28		
Female	30	32	34		
BMI (kg/m^2^)	22.09 ± 2.39	22.01 ± 2.43	22.15 ± 2.31	0.051	0.950^a^
Course of disease (year)	2.08 ± 0.46	2.02 ± 0.51	2.11 ± 0.42	0.596	0.552^a^
Complication					
Stroke	6	9	7	0.567	0.753^b^
Hypertension	9	11	10	0.126	0.938^b^
Diabetes	9	12	8	1.049	0.591^b^
CHA2DS2‐VASc score	3.09 ± 0.45	3.04 ± 0.49	3.11 ± 0.47	0.359	0.699^a^

*Note:* CHA2DS2‐VASc, congestive heart failure, hypertension, age 75, diabetes, prior stroke/transient ischemic attack; VASc, vascular disease, age 65–74 years, sex category (i.e., female gender).

a Statistical tests were compared by one‐way analysis of variance.

^b^ Compared by Chi‐square test.

### 3.2 Comparison of Efficacy

There were 48 effective cases in the Warfarin group, and the total effective rate was 84.21%, which was significantly lower than that in the Rivaroxaban group (60 cases, 98.36%) and the Dabigatran group (60 cases, 96.77%) (*p* < 0.05). However, there was no significant difference in the total effective rate between Rivaroxaban group and Dabigatran group (*p* > 0.05) (Table 2).

**Table 2:** Comparison of efficacy among three groups.

Index	Warfarin group (*n* = 57)	Rivaroxaban group (*n* = 61)	Dabigatran group (*n* = 62)	*χ* ^2^	*p*
Effective (*n*)	48	60	60	11.282	0.004
Ineffective (*n*)	9	1	2		
Total effective rate (%)	84.21	98.36	96.77		

### 3.3 Comparison of Coagulation Function and Liver Function

Before and after treatment, there was no statistical significance in FIB, PT, APTT, and PT among the three groups (all *p* > 0.05) (Table 3). Before and after treatment, there was no significant difference in TbiL, ALP, AST, and ALT among all groups (all *p* > 0.05) (Table 4).

**Table 3:** Comparison of coagulation function among three groups.

Index	Warfarin group (*n* = 57)	Rivaroxaban group (*n* = 61)	Dabigatran group (*n* = 62)	*F*	*p*
FIB (g/L)					
Before the treatment	3.01 ± 0.11	3.02 ± 0.09	3.02 ± 0.12	0.169	0.845
After the treatment	3.04 ± 0.13	3.05 ± 0.12	3.06 ± 0.14	0.350	0.705
PT (s)					
Before the treatment	14.01 ± 0.13	14.02 ± 0.16	14.03 ± 0.14	0.286	0.752
After the treatment	14.76 ± 0.18	14.71 ± 0.13	14.69 ± 0.15	3.221	0.042
APTT (s)					
Before the treatment	38.55 ± 1.87	38.61 ± 1.76	38.62 ± 1.71	0.027	0.973
After the treatment	40.89 ± 2.03	40.83 ± 2.13	40.91 ± 2.09	0.024	0.976
D‐D (mg/L)					
Before the treatment	0.73 ± 0.11	0.71 ± 0.09	0.72 ± 0.07	0.714	0.491
After the treatment	0.58 ± 0.09	0.56 ± 0.07	0.57 ± 0.06	1.079	0.342

Abbreviations: APTT, activated partial thromboplastin time; D‐D, D‐dimer; FIB, fibrinogen; PT, prothrombin time.

**Table 4:** Comparison of liver function among three groups.

Index	Warfarin group (*n* = 57)	Rivaroxaban group (*n* = 61)	Dabigatran group (*n* = 62)	*F*	*p*
ALT (U/L)					
Before the treatment	29.93 ± 2.32	29.89 ± 2.27	29.97 ± 2.43	0.018	0.982
After the treatment	28.79 ± 2.19	28.73 ± 2.23	28.77 ± 2.31	0.011	0.989
AST (U/L)					
Before the treatment	26.77 ± 2.31	26.73 ± 2.29	26.79 ± 2.23	0.011	0.989
After the treatment	25.23 ± 2.18	25.31 ± 2.29	25.25 ± 2.19	0.021	0.979
ALP (U/L)					
Before the treatment	74.49 ± 8.91	74.45 ± 9.03	74.51 ± 8.86	0.001	0.999
After the treatment	71.37 ± 8.36	71.44 ± 8.65	71.41 ± 8.59	0.001	0.999
TbiL (μmol/L)					
Before the treatment	17.09 ± 2.01	17.06 ± 2.11	17.04 ± 2.18	0.008	0.992
After the treatment	15.27 ± 2.38	15.21 ± 2.32	15.31 ± 2.38	0.028	0.972

Abbreviations: ALP, alkaline phosphatase; ALT, alanine transaminase; AST, aspartate aminotransferase; TbiL, total bilirubin.

### 3.4 Comparison of Adverse Reactions

There was no significant difference in the adverse reactions of total incidence of bleeding between Warfarin group (eight cases, 14.04%), Rivaroxaban group (three cases, 4.92%) and Dabigatran group (two cases, 3.23%) (*p* = 0.052) (Table 5).

**Table 5:** Comparison of adverse reactions among three groups.

Index	Warfarin group (*n* = 57)	Rivaroxaban group (*n* = 61)	Dabigatran group (*n* = 62)	*χ* ^2^	*p*
Bleeding events					
Black stool	1	1	1		
Hematuria	1	1	1		
Nosebleed	2	0	0		
Gingival bleeding	3	0	0		
Subcutaneous hemorrhage	1	1	0		
Total bleeding events	8 (14.04%)	3 (4.92%)	2 (3.23%)	5.910	0.052

## 4 Discussion

The results of this study demonstrate that Warfarin, Rivaroxaban, and Dabigatran are effective in preventing thrombotic events in elderly patients with atrial fibrillation, with Rivaroxaban and Dabigatran showing slightly better efficacy. Furthermore, these anticoagulants did not significantly affect liver function, and the incidence of bleeding events was low across all groups, indicating a comparable safety profile. Given these findings, the choice of anticoagulant therapy for elderly patients with atrial fibrillation should prioritize the balance between efficacy and safety, with consideration of patient‐specific factors.

The aging population is a major factor in the rising number of people with atrial fibrillation [15]. The risk of thromboembolic disease in patients with atrial fibrillation is five times higher than that in the general population, and the mortality and disability rate of stroke associated with atrial fibrillation is twice as high as that in patients without atrial fibrillation [16]. Therefore, attention should be paid to whether patients with atrial fibrillation should be treated with the best anticoagulant regimen to reduce the incidence of related stroke, which is of great significance to improve the prognosis of patients. The efficacy and safety of anticoagulation therapy in older patients with atrial fibrillation have been supported by many studies. A retrospective cohort study of 11 760 patients aged 75 years or older with atrial fibrillation receiving oral anticoagulant therapy with Warfarin or nonvitamin K antagonists accounted for 42.4%. The study analyzed the incidence of stroke and intracranial hemorrhage and showed that older patients with atrial fibrillation had a net benefit from anticoagulation therapy [17]. However, in clinical practice, overestimating the bleeding risk of anticoagulant therapy, underestimating the benefits of anticoagulant therapy for stroke prevention and patients' lack of systematic and complete understanding of anticoagulant therapy for atrial fibrillation often interfere with the effective utilization rate of anticoagulant drugs and the standard rate.

Warfarin is one of the most commonly used intermediate‐effect anticoagulants. It plays an anticoagulant role mainly by blocking the synthesis of coagulation factors in liver cells and can effectively inhibit the shedding of thrombosis and prevent the expansion of thrombosis [18]. Warfarin remains the only anticoagulant treatment option for patients with valvular disease‐associated atrial fibrillation and those with severe kidney disease. However, for the anticoagulant treatment of patients with NVAF, the effectiveness of NOAC is at least no worse than that of Warfarin, and the safety is improved. The subgroup analysis of Phase III clinical study of NOAC showed that the reduction of stroke and systemic embolism in the Asian population is better than that in the non‐Asian population [19].

Rivaroxaban, a direct Factor Xa inhibitor, has stable pharmacokinetic properties, does not interact with food and drugs, does not require INR detection, and is an ideal new oral anticoagulant [10]. Dabigatran is a new oral anticoagulant. It is a direct thrombin inhibitor. After oral administration, it is converted into Dabigatran ester in the body, which selectively binds to the specific binding site of thrombin and prevents the decomposition of fibrinogen into fibrin, thus inhibiting thrombus formation [20]. Clinical studies have shown that uninterrupted Dabigatran therapy reduces the risk of stroke in patients with NVAF ablation and has a lower risk of bleeding compared to uninterrupted Warfarin therapy [21]. Compared with Warfarin monotherapy, NOAC monotherapy had a lower risk of bleeding at 45 days, but there was no significant difference in anticoagulant efficacy between NOAC and Warfarin [22]. A meta‐review of the efficacy and safety of nonvitamin K oral antagonists (NOACs) versus Warfarin in elderly patients with atrial fibrillation suggested no significant difference in efficacy or safety between NOAC preparations [23]. The results of this study showed that Warfarin, Rivaroxaban, and Dabigatran were effective in preventing thrombotic events in elderly patients with atrial fibrillation, and rivaroxaban and Dabigatran were more effective Dabigatran.

Possible mechanisms of hypercoagulability in patients with atrial fibrillation include changes in atrial structure and abnormal blood flow, abnormal movement of the irregular atrial wall in atrial fibrillation, resulting in abnormal blood flow and endothelial injury, exposure of subintimal tissues, and release of tissue factors, which activate the coagulation process [24]. It is very important to control the anticoagulant intensity in elderly patients with atrial fibrillation. The results of this study showed that after treatment, FIB, PT, APTT, and PT in all groups were compared (*p* > 0.05), suggesting that Warfarin, Rivaroxaban, and DabigatranDabigatran can effectively regulate coagulation function in elderly patients with atrial fibrillation. Routine clotting function test results are of limited significance for NOAC applications. Unlike Warfarin, which has a narrow therapeutic window, NOAC has a very wide therapeutic window. The precise measurement of NOAC concentration has not yet been widely used in the clinic, but studies have shown that guiding the dosage of NOAC by monitoring the clotting factor activity results does not improve the clinical outcome of patients with atrial fibrillation [25].

Liver and kidney function and duration of administration should be closely monitored. The results of this study showed that after treatment, there were no significant differences in TbiL, ALP, AST, and ALT among all groups (*p* > 0.05), suggesting that Warfarin, Rivaroxaban, and DabigatranDabigatran did not affect liver function in elderly patients with atrial fibrillation. In terms of drug safety, there was no significant difference in the incidence of bleeding events among the three groups and they were all at a low level, suggesting that the safety of the three drugs was similar.

This study offers several notable strengths that enhance its contribution to clinical practice. By providing a comprehensive comparison of Warfarin, Rivaroxaban, and Dabigatran, it delivers valuable insights into the efficacy and safety of these anticoagulant regimens in elderly patients with atrial fibrillation—a population particularly vulnerable to both the condition and the potential complications of anticoagulation therapy. The use of real‐world, retrospective data strengthens the external validity of the findings, making them more applicable to everyday clinical scenarios. Additionally, the study's evaluation of multiple outcome measures, including both efficacy (total effective rate) and safety (adverse reactions, coagulation, and liver function), provides a holistic assessment of patient health. The focused examination of an elderly cohort addresses a critical need for targeted patient care, further underscoring the relevance of the study's findings. There were also some limitations in this study. First, the sample size of this study was small, and there was a lack of multicenter randomized controlled trials. Second, this study lacked follow‐up results of serological indicators in patients after treatment. Finally, data on some other factors that may contribute to thrombotic events are missing, such as smoking. The result of this study still needs to be fully validated by long‐term follow‐up studies involving a larger sample of patients.

## 5 Conclusion

Warfarin, Rivaroxaban, and Dabigatran can effectively prevent thrombosis in elderly patients with atrial fibrillation, and rivaroxaban and dabigatran have better effects. The three drugs had similar and low rates of bleeding events and did not have significant effects on coagulation and liver function.

## Author Contributions

Cheng Chen and Qian Zhou contributed to the conception and design of the study. All authors participated in the clinical practice, including diagnosis, treatment, consultation, and follow‐up of patients. Qing Tian and Chong Cheng contributed to the acquisition of data. Xixin Ji, Mengyun Feng, and Huiqun Tan contributed to the analysis of data. Cheng Chen wrote the manuscript. Qian Zhou and Qing Tian revised the manuscript. All authors approved the final version of the manuscript.

## Ethics Statement

This study was approval by the ethical review committee of Huangshi Central Hospital. Informed consent was waived due to the retrospective nature of the study. This study was conducted in compliance with the Helsinki Declaration.

## Consent

The authors have nothing to report.

## Conflicts of Interest

The authors declare no conflicts of interest.

## Data Availability

The data that support the findings of this study are available on request from the corresponding author. The data are not publicly available due to privacy or ethical restrictions.
